# Two-Dimensional Piezoresistive Response and Measurement of Sensitivity Factor of Polymer-Matrix Carbon Fiber Mat

**DOI:** 10.3390/polym12123072

**Published:** 2020-12-21

**Authors:** Min Wu, Li Huang, Xiaoyu Zhang, Jianzhong Chen, Yong Lv

**Affiliations:** 1 School of Science, Wuhan University of Technology, Wuhan 430070, China; wum@whut.edu.cn; 2 Hubei Key Laboratory of Theory and Application of Advanced Materials Mechanics, Wuhan University of Technology, Wuhan 430070, China; zxysandy@126.com (X.Z.); cjzwhut@163.com (J.C.); lvyonghl@163.com (Y.L.)

**Keywords:** polymer-matrix, carbon fiber mat, orthogonal strain, piezoresistive sensitivity factor, piezoresistive effect

## Abstract

Based on the piezoresistive effect, the piezoresistive constitutive relation of a carbon fiber mat under orthogonal strain was deduced. Considering the Poisson effect, the piezoresistive responses and measurement of the sensitivity factor of a polymer-matrix carbon fiber mat under bidirectional strain were studied by a two-times uniaxial tension loading method in different directions, which was pasted in the center area of a cruciform aluminum substrate. The relations between the resistance change rate and the orthogonal strains were established, the reasonability of which was confirmed by comparison with the experimental results. The results show that the longitudinal piezoresistive sensitivity factor *C*_11_ is 21.55, and the lateral piezoresistive sensitivity factor *C*_12_ is 24.15. Using these factors, the resistance change rate of another polymer-matrix carbon mat was predicted, which was made by the same technique, and the error between the predicted and the experimental results was 1.3% in the longitudinal direction and 6.1% in the lateral direction.

## 1. Introduction

Carbon fiber materials are widely used in military, aerospace and other fields due to their high strength, light weight, fatigue resistance, corrosion resistance and other excellent properties. Since the piezoresistive responses of carbon fiber-reinforced plastics (CFRP) were discovered in 1980s, the piezoresistive responses characteristics of carbon fiber material made by different methods (e.g., carbon fiber bundle [1], single carbon fiber epoxy composites [2], continuous carbon fiber prepreg [3]), and under different loading modes, for instance, unidirectional cyclic loading [4,5], uniaxial tension [6] and unidirectional compression [7], have been studied by the majority of scholars. Utilizing the piezoresistive responses characteristics of carbon fiber materials, when carbon fiber material was embedded in concrete it could be used to detect internal defects and damage [8,9]; if it was laid on the surface of a concrete beam, called a carbon fiber smart layer, it could be used for surface crack detection or beam deformation monitoring [10,11]. In order to establish the mapping relationship between strain and resistance of a carbon fiber smart layer, the existing research mainly focuses on the behaviors of a carbon fiber monofilament [12], carbon fiber bundle [13] and polymer-matrix carbon fiber mat [14].

Regarding the piezoresistive constitutive research of a polymer-matrix carbon fiber mat, the joint effect of multi-directional strain has not been considered in existing reports. The relationship between resistance change (rate) and unidirectional stress or strain was established, and did not involve complex strain states. However, in the actual project, forced components are often in a complex stress state, and the piezoresistive constitutive derived from the unidirectional strain field is no longer unconvincing. Therefore, it is of great significance to study the piezoresistive response of a polymer-matrix carbon fiber mat under complex stress or strain, which lays a foundation for the application of a carbon fiber mat in the actual structure detection.

In this paper, based on piezoresistive theory, the constitutive relations of piezoresistive under bidirectional strain was derived. In order to measure the resistance response of a carbon fiber mat under orthogonal strain, and to avoid the errors caused by the differences between different samples, it was measured by two-times unidirectional tensile experiments in different directions, which were attached to a cruciform specimen of aluminum. According to the experimental results, the resistance sensitive factor was solved, and the constitutive relation of the polymer-matrix carbon mat was established and verified.

## 2. Theory of Piezoresistive Model

The change of the resistivity of solid material induced by the load is called the piezoresistive effect. For three-dimensional materials, the piezoresistive coefficient matrix is a square matrix of 6 × 6, and Equation (1) is the resistivity change rate caused by stresses [15].

(1) $$\left[\begin{array}{l} {\left(\frac{\Delta \rho }{\rho_{0}}\right)}_{1}\\ {\left(\frac{\Delta \rho }{\rho_{0}}\right)}_{2}\\ {\left(\frac{\Delta \rho }{\rho_{0}}\right)}_{3}\\ {\left(\frac{\Delta \rho }{\rho_{0}}\right)}_{4}\\ {\left(\frac{\Delta \rho }{\rho_{0}}\right)}_{5}\\ {\left(\frac{\Delta \rho }{\rho_{0}}\right)}_{6} \end{array}\right]=\left[\begin{array}{l} \pi_{11}\;\pi_{12}\;\pi_{13}\;\pi_{14}\;\pi_{15}\;\pi_{16}\\ \pi_{21}\;\pi_{22}\;\pi_{23}\;\pi_{24}\;\pi_{25}\;\pi_{26}\\ \pi_{31}\;\pi_{32}\;\pi_{33}\;\pi_{34}\;\pi_{35}\;\pi_{36}\;\\ \pi_{41}\;\pi_{42}\;\pi_{43}\;\pi_{44}\;\pi_{45}\;\pi_{46}\;\\ \pi_{51}\;\pi_{52}\;\pi_{53}\;\pi_{34}\;\pi_{55}\;\pi_{56}\;\\ \pi_{61}\;\pi_{62}\;\pi_{63}\;\pi_{64}\;\pi_{65}\;\pi_{66}\; \end{array}\right]\left[\begin{array}{l} \sigma_{1}\\ \sigma_{2}\\ \sigma_{3}\\ \sigma_{4}\\ \sigma_{5}\\ \sigma_{6} \end{array}\right]$$

where, $\sigma_{1}$,$\sigma_{2}$, $\sigma_{3}$, $\sigma_{4}$, $\sigma_{5}$, $\sigma_{6}$ are the stress components describing the stress state of solid material; $\sigma_{1}$, $\sigma_{2}$, $\sigma_{3}$ are independent normal stresses and $\sigma_{4}$, $\sigma_{5}$,$\sigma_{6}$ are independent shear stresses.

When the three-dimensional solid material is an isotropic material, $\pi_{11}=\pi_{22}=\pi_{33}$; $\pi_{12}=\pi_{13}=\pi_{23}=\pi_{21}=\pi_{31}=\pi_{32}$; $\pi_{44}=\pi_{55}=\pi_{66}$. meanwhile, the piezoresistive effect has the following characteristics:

(1) It is impossible for shear stress to produce positive piezoresistive effect,
  $$\pi_{14}=\pi_{15}=\pi_{16}=\pi_{24}=\pi_{25}=\pi_{26}=\pi_{34}=\pi_{35}=\pi_{36}=0.$$
(2) Normal stress cannot produce shear piezoresistive effect,
  $$\pi_{41}=\pi_{51}=\pi_{61}=\pi_{42}=\pi_{52}=\pi_{62}=\pi_{43}=\pi_{53}=\pi_{63}=0.$$
(3) Shear stress can only produce piezoresistive effect in the plane which acts on,
  $$\pi_{45}=\pi_{46}=\pi_{34}=\pi_{56}=\pi_{64}=\pi_{65}=0.$$

Furthermore, for the plane stress state, $\sigma_{3}=0$, $\sigma_{5}=0$, $\sigma_{6}=0$, thus the piezoresistive equation can be expressed by Equation (2).

(2) $$\left\{\begin{array}{l} {\left(\frac{\Delta \rho }{\rho_{0}}\right)}_{1}=\pi_{11}\sigma_{1}+\pi_{12}\sigma_{2}\\ {\left(\frac{\Delta \rho }{\rho_{0}}\right)}_{2}=\pi_{12}\sigma_{1}+\pi_{11}\sigma_{2}\\ {\left(\frac{\Delta \rho }{\rho_{0}}\right)}_{4}=\pi_{44}\sigma_{4} \end{array}\right.$$

Combined with Hooke’s law in the plane stress state,

(3) $$\left[\begin{array}{c} \sigma_{1}\\ \sigma_{2}\\ \sigma_{4} \end{array}\right]=\frac{E}{\left(1-\mu^{2}\right)}\left[\begin{array}{ccc} 1 & \mu & 0\\ \mu & 1 & 0\\ 0 & 0 & \frac{1-\mu }{2} \end{array}\right]\left[\begin{array}{c} \varepsilon_{1}\\ \varepsilon_{2}\\ \varepsilon_{4} \end{array}\right]$$

Equation (2) can be expressed as the rate of resistivity change caused by strains, as shown in Equation (4).

(4) $$\left\{\begin{array}{l} {\left(\frac{\Delta \rho }{\rho_{0}}\right)}_{1}=C_{11}\varepsilon_{1}+C_{12}\varepsilon_{2}\\ {\left(\frac{\Delta \rho }{\rho_{0}}\right)}_{2}=C_{12}\varepsilon_{1}+C_{11}\varepsilon_{2}\\ {\left(\frac{\Delta \rho }{\rho_{0}}\right)}_{4}=C_{44}\varepsilon_{4} \end{array}\right.$$

where, $C_{11}=\frac{E(\pi_{11}+\mu \pi_{12})}{1-\mu^{2}}$, $C_{12}=\frac{E(\mu \pi_{11}+\pi_{12})}{1-\mu^{2}}$, $C_{44}=\frac{E\pi_{44}}{21+\mu }$ respectively represent the longitudinal piezoresistive sensitivity factor, the lateral piezoresistive sensitivity factor, and the shear piezoresistive sensitivity factor; *µ* is Poisson’s ratio and *E* is the elastic modulus.

## 3. Experimental Research

### 3.1. Raw Material

The PAN-based carbon fiber mat of 0.05 mm thickness was manufactured by Pengyuan fiberglass Products Co. Ltd. The raw material parameters of the mat are shown in Table 1. Conductive silver paint for electrodes adhering to the carbon fiber mat was manufactured by SPI Corporation. Other materials: 0.1 mm diameter copper core wire.

### 3.2. Specimens Preparation

**Figure 1:**
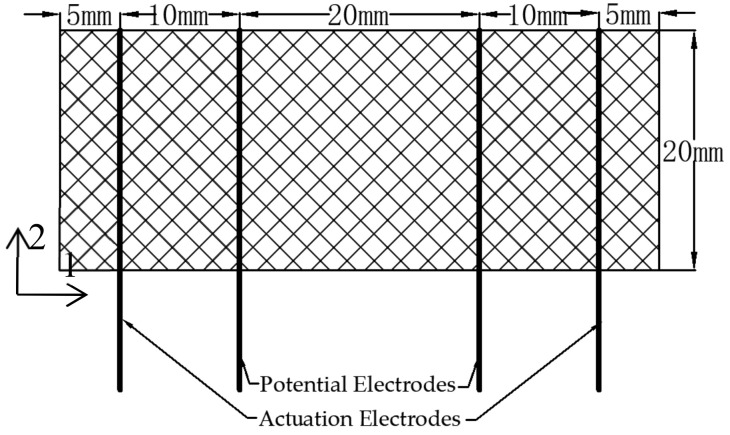
Schematic diagram of electrode layout.

The carbon fiber mat was cut to the size of 50 mm × 20 mm. In order to measure the resistance of the carbon fiber mat, the four-electrode method was used to eliminate the contact resistance between the electrodes and the carbon fiber mat. Four copper wires were bonded to the carbon fiber mat at predetermined positions with conductive silver glue to ensure that the distance between the actuation electrodes for current excitation was 40 mm, and the distance between the potential electrodes for voltage acquisition was 20 mm, as shown in Figure 1. It was assumed that the length direction of the carbon fiber mat was the 1st-direction, and the width direction was the 2nd-direction.

**Figure 2:**
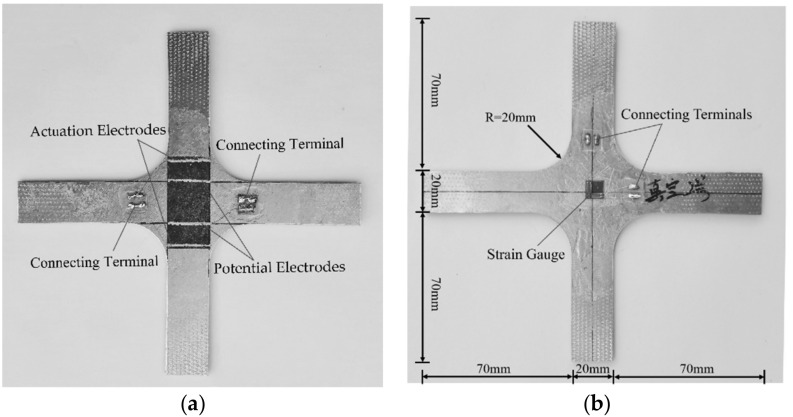
Front and back of the specimen: (**a**) polymer-matrix carbon mat attachment position on the front; (**b**) strain gauge attachment position on the back.

The process of polymer-matrix carbon fiber mat was as follows: the E-44 epoxy resin (product of Yueyang Petrochemical Complex), and 2-ethyl-4-methylimidazole were mixed at a mass ratio of 100:10, and then 4% glycidyl phenyl ether was added for dilution. The 3 mm-thickness insulated cruciform aluminum substrate with width of 20 mm was brushed the diluted epoxy resin. The carbon fiber mat with electrodes was pasted on the central position of the aluminum substrate, ensuring that the distance between the potential electrodes coincides with the width of the aluminum substrate. Then the diluted epoxy resin was brushed again on the upper surface of the mat. Finally, the specimen was covered with peel ply, and placed into a vacuum bag for pressure curing. After 7 days’ solidification at room temperature, the specimen was demoulded. Two perpendicular strain gauges were pasted on the back of the aluminum substrate, as illustrated in Figure 2.

### 3.3. The Experimental Process

**Figure 3:**
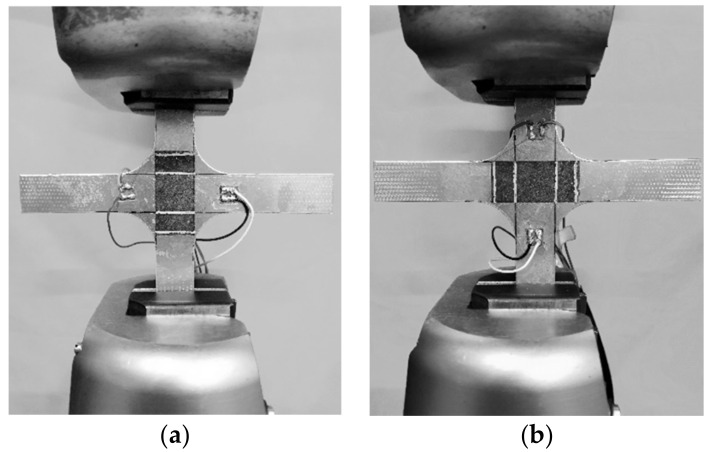
Device diagram of loading: (**a**) loading along the 1st-direction; (**b**) loading along the 2nd-direction.

Two-times uniaxial loading in different directions was carried out on an Instron 5882 material test machine. The loading speed was 2000 N/min and the maximum load was 5000 N under force control, as shown in Figure 3. During loading, Keithley 6221 was used to provide a constant 1 mA direct current (DC) to the actuation electrodes, and the voltage between the potential electrodes was collected by a Keithley 2700 with the acquisition frequency of 1 Hz. The resistance of the carbon fiber mat was calculated by Ohm’s law, that is, the voltage divided by current.

## 4. Results and Discussion

### 4.1. Experimental Results

**Figure 4:**
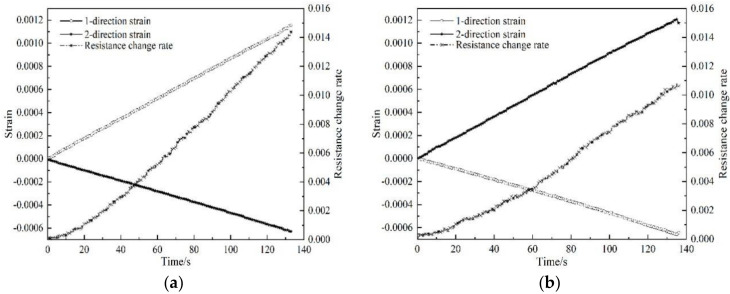
Test curve of sample No. 1: (**a**) loading along the 1st-direction; (**b**) loading along the 2nd-direction.

**Figure 5:**
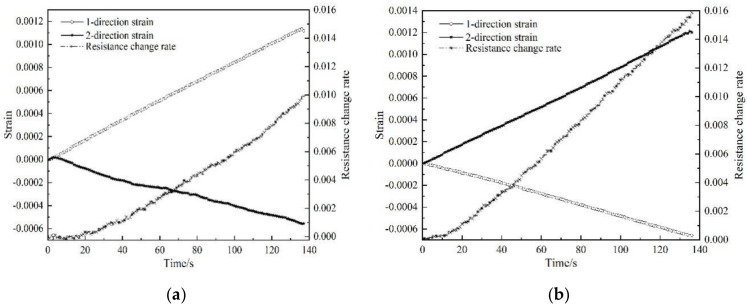
Test curve of sample No. 2: (**a**) loading along the 1st-direction; (**b**) loading along the 2nd-direction.

Two groups of tests were conducted on the same batch of samples. The relationship between the resistance response of the polymer-matrix carbon fiber mat and the two orthogonal strains is as shown in Figure 4 and Figure 5, where the resistance change rate is the ratio of the resistance change to the original resistance.

**Figure 6:**
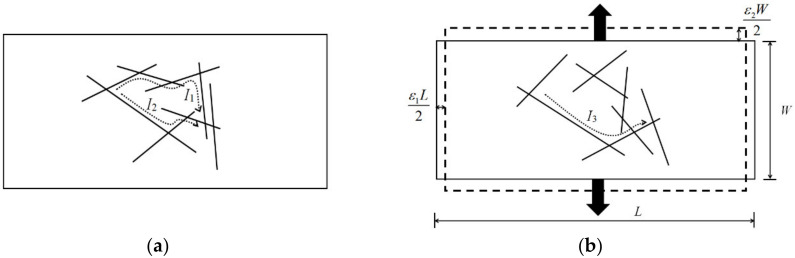
Schematic diagram of the conductive network of the carbon fiber mat: (**a**) before loading; (**b**) after loading.

The experimental results show that when the loading direction is consistent with the 1st-direction of the carbon fiber mat, the resistance change rate is greatly affected by the 1st-direction strain; when the loading direction is consistent with the 2nd-direction, due to the Poisson effect, strain in the 1st-direction is negative, and the resistance change rate still increases, which indicates the positive piezoresistive effect. This phenomenon is caused by the change of the gap between the fibers in the carbon fiber mat. During loading, the force caused the fibers to rotate or translate and made some overlapped fibers fall off, resulting in the change of the number of conductive channels. As shown in Figure 6, when the loading direction is consistent with the 2nd-direction, some fibers in the carbon fiber mat change before and after loading. The two conductive paths before loading form a parallel connection, and the conductive path is reduced to one after loading. According to the calculation theory of series and parallel resistance, the resistance will increase. This phenomenon demonstrates that the sensing characteristics of the carbon fiber mat are determined by strains in two directions, and the Poisson effect has a non-negligible influence on the sensing characteristics of the carbon fiber mat.

### 4.2. Solution of Piezoresistivity Sensitivity Factor

Because the carbon fiber mat is very thin and tightly pasted on the surface of the aluminum substrate, it can be considered that the strain of the two materials is the same. For the carbon fiber mat, the resistance expression is shown in Equation (5):

(5) $$R=\rho \frac{L}{W}$$

where, ρ is the surface resistivity of the carbon fiber mat, *L* is the length and *W* is the width of the carbon fiber mat between the potential electrodes.

From Equation (5), the resistance change rate in the piezoresistivity can be derived, as shown in Equation (6):

(6) $$\frac{\Delta R}{R_{0}}=\frac{\Delta \rho }{\rho_{0}}+\frac{\Delta L}{L}-\frac{\Delta W}{W}=\frac{\Delta \rho }{\rho_{0}}+\varepsilon_{1}-\varepsilon_{2}$$

Combining Equation (6) with the first two formulas of Equation (4), the relationship between the change rate of resistance and strain of the carbon fiber mat can be obtained, as shown in Equation (7):

(7) $$\left\{\begin{array}{l} {\left(\frac{\Delta R}{R_{0}}\right)}_{1}=(C_{11}+1)\varepsilon_{1}+(C_{12}-1)\varepsilon_{2}\\ {\left(\frac{\Delta R}{R_{0}}\right)}_{2}=(C_{12}-1)\varepsilon_{1}+(C_{11}+1)\varepsilon_{2} \end{array}\right.$$

According to the experimental results in Figure 4 and Figure 5, the change rate of resistance of the carbon fiber mat corresponding to the maximum strain is substituted into Equation (7), respectively, and the piezoresistive sensitivity factor of the carbon fiber mat under the action of bidirectional strain could be calculated, as shown in Table 2.

The piezoresistive constitutive of the polymer-matrix carbon fiber mat under the action of bidirectional strain can be obtained, as shown in Equation (8):

(8) $$\left\{\begin{array}{l} {\left(\frac{\Delta R}{R_{0}}\right)}_{1}=22.55\varepsilon_{1}+23.15\varepsilon_{2}\\ {\left(\frac{\Delta R}{R_{0}}\right)}_{2}=23.15\varepsilon_{1}+22.55\varepsilon_{2} \end{array}\right.$$

### 4.3. Piezoresistive Constitutive Verification

**Figure 7:**
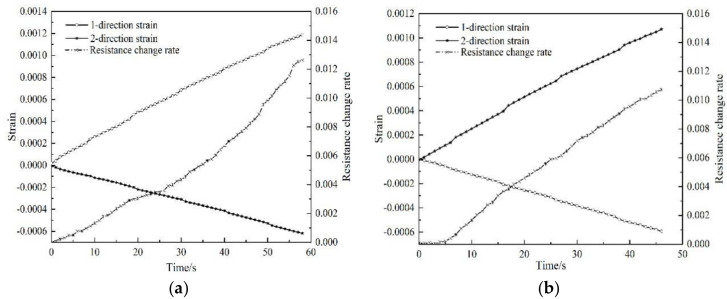
Test curve of sample No. 3: (**a**) loading along the 1st-direction; (**b**) loading along the 2nd-direction.

In order to verify the accuracy of the piezoresistive constitutive relation, a similar force resistance experiment was performed on the No. 3 sample. The experimental results are shown in Figure 7:

The resistance change rate of the No. 3 polymer-matrix carbon fiber mat sample is predicted by Equation (8), and the specific results are shown in Table 3. Obviously, the predicted resistance change rate ${\left(\frac{\Delta R}{R}\right)}_{\mathrm{pre}}$ is very close to the experimental resistance change rate ${\left(\frac{\Delta R}{R}\right)}_{\exp}$. The results prove that this constitutive relation can better reflect the resistance response law of the carbon fiber mat under orthogonal strains.

## 5. Conclusions

(1) Based on piezoresistance theory, the constitutive model of the polymer-matrix carbon fiber mat under plane stress state was deduced in this paper, and the response law of its resistance under bidirectional strains was investigated. Combined with the experimental results, the piezoresistance sensitivity factor of the polymer-matrix carbon fiber mat was obtained, and the piezoresistance constitutive relationship in the orthogonal strain state was established. The rationality of the piezoresistance relationship was verified by comparing it with the experimental results.
(2) In this study, the shear piezoresistance sensitivity factor of the carbon fiber mat could not be measured. Therefore, further research is needed in the future.

## Figures and Tables

**Table 1:** Raw material parameters of PAN-based carbon fiber mat.

Name	Specification (g/m^2^)	Organic Matter Content (%)	Fiber Diameter (μm)	Water Content (%)
Parameters	10	6–12	7.02 ± 2	≤0.5

**Table 2:** Resistance sensitivity factor of carbon fiber mat under bidirectional strain.

Groups	Longitudinal Piezoresistive Sensitivity Factor/*C*_11_	Lateral Piezoresistive Sensitivity Factor/*C*_12_
No. 1	23.6	23.3
No. 2	19.5	25.0
Average value	21.55	24.15

**Table 3:** Predicted and experimental values of resistance change rate of polymer-matrix carbon mat.

Loading Direction	ε_1_ × (10^−6^)	ε_2_ × (10^−6^)	${\left(\frac{\Delta R}{R}\right)}_{\exp}$	${\left(\frac{\Delta R}{R}\right)}_{\mathrm{pre}}$	Error
1-direction	1186.1	−618.1	0.0126036	0.0124375	1.3%
2-direction	−593.5	1071.7	0.0107288	0.0114264	6.1%
